# Predicting survival of patients with bone metastasis of unknown origin

**DOI:** 10.3389/fendo.2023.1193318

**Published:** 2023-11-06

**Authors:** Ying Ren, Shengjun Qian, Guoping Xu, Zhenhai Cai, Ning Zhang, Zhan Wang

**Affiliations:** ^1^ Department of Nursing, The Second Affiliated Hospital of Zhejiang University School of Medicine, Hangzhou, China; ^2^ Department of Orthopedic Surgery, The Second Affiliated Hospital, Zhejiang University School of Medicine, Hangzhou, China; ^3^ Orthopedics Research Institute of Zhejiang University, Hangzhou, China; ^4^ Key Laboratory of Motor System Disease Research and Precision Therapy of Zhejiang Province, Hangzhou, China; ^5^ Zhejiang Provincial Clinical Medical Research Center for Motor System Diseases, Hangzhou, China; ^6^ International Chinese Musculoskeletal Research Society, Hangzhou, China; ^7^ Department of Orthopedics Surgery, The Second Affiliated Hospital of Jiaxing University, Jiaxing, China

**Keywords:** bone metastasis, unknown origin, clinical characteristics, survival, risk factor

## Abstract

**Purpose:**

Bone metastasis of unknown origin is a rare and challenging situation, which is infrequently reported. Therefore, the current study was performed to analyze the clinicopathologic features and risk factors of survival among patients with bone metastasis of unknown origin.

**Patients and methods:**

We retrospectively analyzed the clinical data for patients with bone metastasis of unknown origin between 2010 and 2016 based on the Surveillance, Epidemiology, and End Results (SEER) database. Overall survival (OS) and cancer-specific survival (CSS) were first analyzed by applying univariable Cox regression analysis. Then, we performed multivariable analysis to confirm independent survival predictors.

**Results:**

In total, we identified 1224 patients with bone metastasis of unknown origin for survival analysis, of which 704 males (57.5%) and 520 females (42.5%). Patients with bone metastasis of unknown origin had a 1-year OS rate of 14.50% and CSS rate of 15.90%, respectively. Race, brain metastasis, liver metastasis, radiotherapy, and chemotherapy were significant risk factors of OS on both univariable and multivariable analyses (p <0.05). As for CSS, both univariable and multivariable analyses revealed that no brain metastasis, no liver metastasis, radiotherapy, and chemotherapy were associated with increased survival (p <0.05).

**Conclusion:**

Patients with bone metastasis of unknown origin experienced an extremely poor prognosis. Radiotherapy and chemotherapy were beneficial for prolonging the survival of those patients.

## Introduction

Bone is one of the most common organs in cancer metastasis, especially in lung, breast, and prostate cancer (1, 2). Once bone metastasis is developed, patients’ survival and quality of life will be significantly declined (3). The prognosis of bone metastases from different tumor types varies greatly. Therefore, to identify the primary pathologic type of bone metastasis is the key to the treatment of such patients. However, up to 30% of patients present with bone metastasis of unknown origin after detailed investigations (4). The spine is reported to be the most common site of bone metastasis of unknown origin, followed by the pelvis and long bones (5). Some studies showed that patients with bone metastasis of unknown origin had a poor outcome with a mean survival ranging from 3 to 12 months from diagnosis (6–9). The characteristics and survival of patients with bone metastasis of a certain known origin have been widely reported. However, few studies have been reported on the characteristics and risk factors affecting the prognosis in patients with bone metastasis of unknown origin. Additionally, effective treatments for such patients remain unknown.

To date, there was no large-sample studies to analyze the prognosis of patients with bone metastasis of unknown origin. The different characteristics and survival of patients with bone metastasis of unknown origin still need to be elucidated. In order to provide an insight into the bone metastasis of unknown origin, we used the Surveillance, Epidemiology, and End Results (SEER) database to reveal the clinicopathologic features and prognostic factors. Our findings may provide timely interventions for those patients to improve their survival.

## Materials and methods

### Patient population

Patients with bone metastasis of unknown origin were retrieved between 2010 and 2016 from the SEER database. This study used the case-listing session on the SEER*Stat version 8.3.9 software to extract the clinical data. We selected patients with unknown origin by using the Primary Site - labeled “C80.9-Unknown primary site”. Meanwhile, we set the SEER Combined Mets at DX-bone (2010+) to be YES. Patients without pathological diagnosis were excluded. Medical ethics review was not required in this study because clinical data in the present study were extracted from a public database.

Race, gender, age at diagnosis, histopathological type, brain metastasis, liver metastasis, lung metastasis, radiotherapy, chemotherapy, marital status, vital status, survival time, and cause of death were included for analysis. Overall survival (OS) and cancer-specific survival (CSS) were defined as the time from diagnosis till death due to any cause and due to the cancer, respectively.

### Statistical analysis

All statistical and descriptive analysis were performed by using the SPSS 22.0 software. Univariable Cox regression models were used to investigate the potential risk factors for prognosis. Significant risk factors from univariable analysis were incorporated for multivariable Cox regression analysis. Meanwhile, hazard ratio (HR) and its 95% confidence interval (95% CI) were recorded in univariable and multivariable analyses. The Kaplan-Meier method was applied to draw survival curves, and the Log-rank test was performed to compare the survival difference. The difference was statistically significant with bilateral p value less than 0.05.

## Results

### Baseline characteristics

The detailed patient clinical characteristics are summarized in **Table 1**. In total, 1224 cases who met the eligibility criteria were included in this study.

**Table 1:** Baseline characteristics of 1224 patients with bone metastasis of unknown origin.

Variable	Value
Race	
White	1014 (82.8%)
Black	135 (11.0%)
Others	75 (6.1%)
Gender	
Female	520 (42.5%)
Male	704 (57.5%)
Age at diagnosis	
≤60	319 (26.1%)
>60	905 (73.9%)
Histology group	
Epithelial neoplasms, NOS	373 (30.5%)
Squamous cell neoplasms	114 (9.3%)
Adenomas and adenocarcinomas	593 (48.4%)
Others	144 (11.8%)
Brain metastasis	
No	889 (72.6%)
Yes	144 (11.8%)
Unknown	191 (15.6%)
Liver metastasis	
No	512 (41.8%)
Yes	568 (46.4%)
Unknown	144 (11.8%)
Lung metastasis	
No	606 (49.5%)
Yes	439 (35.9%)
Unknown	179 (14.6%)
Radiotherapy	
Yes	417 (34.1%)
No	807 (65.9%)
Chemotherapy	
Yes	350 (28.6%)
No	874 (71.4%)
Marital status	
Married	615 (50.2%)
Others	548 (44.8%)
Unknown	61 (5.0%)
Dead	
Yes	1034 (84.5%)
No	190 (15.5%)
**1-year OS rate**	14.50%
**1-year CSS rate**	15.90%

OS, overall survival; CSS, cancer-specific survival.

There were 704 males (57.5%) and 520 females (42.5%). Their mean age was 68 years (range, 3-100 years). We divided the age into two groups: ≤60 years (26.1%), and >60 years (73.9%). About four fifths (82.8%) of patients were white race. Adenomas and adenocarcinomas type was the main histological type, accounting for 48.4% of all cases, followed by epithelial neoplasms, NOS type. In terms of other organ metastasis, 144 (11.8%) cases had brain metastasis, 568(46.4%) had liver metastasis, and 439(35.9%) had lung metastasis. Overall, 34.1% of patients underwent radiotherapy, and 28.6% of patients had chemotherapy. There were 615(50.2%) patients with married status, 548(44.8%) patients with other marital status, and 61(5.0%) patients with unknown marital status. The 1-year OS and CSS rates of patients were 14.5% and 15.9%, respectively.

### Univariable Cox regression analysis

The detailed univariable analysis results of patients with bone metastasis of unknown origin were showed in **Table 2**. No significance on both OS and CSS were observed in terms of gender, histological type, and marital status. Patients with other races were significantly associated with better OS but not CSS. Age >60 years was independently associated with worse survival. Other distant metastases significantly decreased OS and CSS. Patients underwent radiotherapy and chemotherapy had better OS and CSS.

**Table 2:** Univariate Cox analysis of variables in patients with bone metastasis of unknown origin.

Variable	OS		CSS	
	HR (95% CI)	*P*	HR (95% CI)	*P*
Race				
White	1		1	
Black	0.888 (0.732-1.078)	0.23	0.901 (0.716-1.134)	0.377
Others	0.746 (0.565-0.985)	0.039	0.758 (0.548-1.048)	0.093
Gender				
Female	1		1	
Male	1.029 (0.909-1.165)	0.648	0.966 (0.834-1.118)	0.642
Age at diagnosis				
≤60	1		1	
>60	1.203 (1.045-1.384)	0.01	1.196 (1.016-1.407)	0.032
Histology group				
Epithelial neoplasms, NOS	1		1	
Squamous cell neoplasms	0.898(0.715-1.129)	0.357	0.882(0.661-1.175)	0.39
Adenomas and adenocarcinomas	1.043 (0.906-1.202)	0.554	1.050(0.888-1.242)	0.566
Others	0.991 (0.805-1.220)	0.93	0.926 (0.714-1.200)	0.56
Brain metastasis				
No	1		1	
Yes	1.218(1.009-1.470)	0.04	1.259(1.015-1.562)	0.036
Liver metastasis				
No	1		1	
Yes	1.306(1.145-1.489)	<0.001	1.387(1.185-1.624)	<0.001
Lung metastasis				
No	1		1	
Yes	1.204(1.052-1.378)	0.007	1.213(1.033-1.424)	0.019
Radiotherapy				
Yes	1		1	
No	1.434(1.259-1.634)	<0.001	1.500(1.281-1.756)	<0.001
Chemotherapy				
Yes	1		1	
No	2.048(1.778-2.358)	<0.001	2.058(1.738-2.438)	<0.001
Marital status				
Married	1		1	
Others	1.041 (0.919-1.180)	0.526	1.031 (0.887-1.198)	0.691

### Multivariable Cox regression analysis

**Figure 1:**
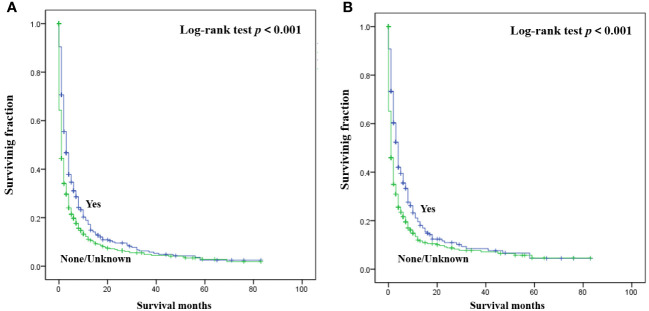
Kaplan-Meier method estimated OS **(A)** and CSS **(B)** in patients with bone metastasis of unknown origin stratified by radiotherapy. (OS, overall survival; CSS, cancer-specific survival).

**Figure 2:**
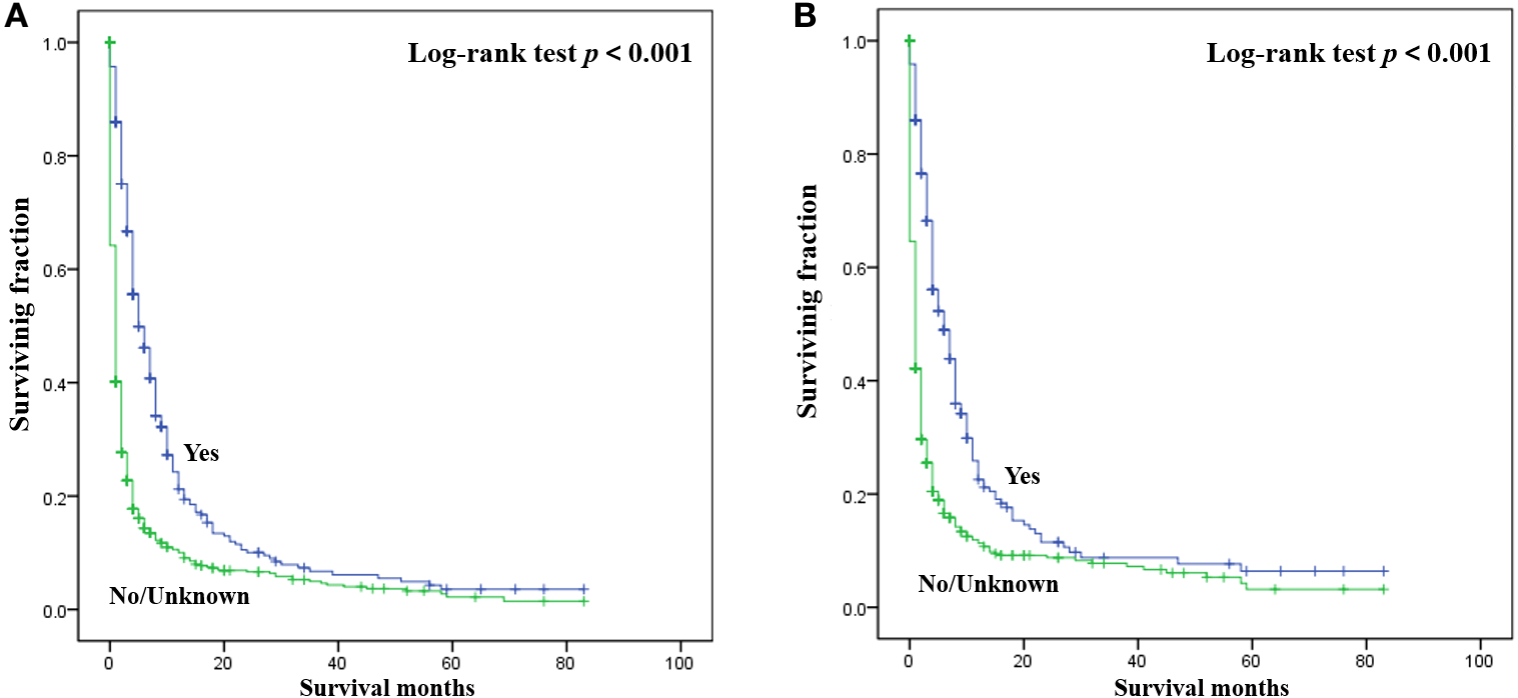
Kaplan-Meier method estimated OS **(A)** and CSS **(B)** in patients with bone metastasis of unknown origin stratified by chemotherapy. (OS, overall survival; CSS, cancer-specific survival).

The detailed multivariable analysis results of patients with bone metastasis of unknown origin were showed in **Table 3**. Age at diagnosis and lung metastasis were no longer significant risk factors for prognosis. On multivariable analysis of OS, white race, brain metastasis, liver metastasis, no radiotherapy, and no chemotherapy were significantly associated with decreased survival. On multivariable analysis of CSS, brain metastasis, liver metastasis, no radiotherapy, and no chemotherapy were significantly associated with decreased survival. Kaplan-Meier survival analysis stratified by radiotherapy and chemotherapy were shown in **Figures 1**, **2**, respectively.

**Table 3:** Multivariate Cox analysis of variables in patients with bone metastasis of unknown origin.

Variable	OS		CSS	
	HR (95% CI)	*P*	HR (95% CI)	*P*
Race				
White	1		-	
Black	0.860(0.708-1.046)	0.132	-	-
Others	0.656 (0.496-0.868)	0.003	-	-
Age at diagnosis				
≤60	1		1	
>60	1.108 (0.960-1.280)	0.161	1.082 (0.915-1.280)	0.357
Brain metastasis				
No	1		1	
Yes	1.292(1.063-1.570)	0.01	1.336(1.070-1.669)	0.011
Liver metastasis				
No	1		1	
Yes	1.267(1.096-1.463)	0.001	1.336(1.125-1.587)	0.001
Lung metastasis				
No	1		1	
Yes	1.115(0.965-1.289)	0.14	1.094(0.922-1.299)	0.301
Radiotherapy				
Yes	1		1	
No	1.316(1.147-1.509)	<0.001	1.327(1.124-1.567)	0.001
Chemotherapy				
Yes	1		1	
No	2.133(1.843-2.469)	<0.001	2.088(1.751-2.489)	<0.001

## Discussion

Bone is the third most common metastatic site following the liver and lung (10). Cancer of unknown origin refers to malignancies, where metastases are histologically confirmed, but where no primary site can be identified on the basis of a comprehensive clinical and imaging evaluation (11–13). To our knowledge, the current study is the largest population-based study to explore the clinical features and survival predictors for patients with bone metastasis of unknown origin. With the progress of diagnosis and treatment technology, the proportion of unknown primary tumors in metastatic tumors has steadily declined (13). However, those patients experienced extremely poor prognosis. In the present study, we first defined the clinicopathological features and prognosis of this special population. Our study found that the 1-year OS and CSS rates for patients with bone metastasis of unknown origin were 14.5% and 15.9%, respectively. Therefore, accurate assessment of patients’ prognostic risk factors is helpful to improve their prognosis and assist clinicians to make reasonable treatment decisions. More importantly, this study provides evidence for future treatment guidelines for such patients.

It is worth mentioning that our analysis found that gender and age were not independent risk factors for survival. However, many previous studies on bone metastasis have found that they indeed correlated with the patient’s prognosis (14, 15). This may be due to the diverse pathologic types of the primary lesion. In terms of race, significant difference was observed in OS but not in CSS, which was not in line with other studies on bone metastasis (16, 17). Further researches are needed to clarify this risk factor. Regarding the tumor histopathology, adenomas and adenocarcinomas type accounted for almost half of all cases, but it was not an independent predictor of survival. It seems that the histopathologic type of the tumor has little effect on prognosis in patients with bone metastasis of unknown origin.

Generally, once a tumor develops distant metastasis in one organ, it may accelerate metastasis in other organs. Interestingly, bone metastasis combined with lung metastasis do not result in a worse prognosis in these patients, whereas bone metastasis combined with brain or liver metastases do. Ya Qin et al. (18) also reported the similar results among esophageal cancer patients with bone metastasis. It seems that there is a homologous relationship between bone metastasis and lung metastasis. Most studies have found that lung, liver and brain metastases are closely related to the prognosis of patients (19–21). Our study revealed that marital status was not associated with survival. However, some researches demonstrated that marital status was an independent prognostic factor for survival among patients with bone metastasis of known origin (19, 22).

A standard treatment for patients with bone metastasis of unknown origin has not yet been developed. Treatments for vertebral lesions include surgical excision and non-surgical management (10, 23). Our multivariable analyses revealed that radiotherapy and chemotherapy were significantly correlated with prognosis, which provides an optimal strategy for treating them. Chemotherapy was also an independent survival predictor for primary bone tumors, such as osteosarcoma (24). Various chemotherapeutic regimens are reported in the treatment of malignant primary bone tumors of the spine. However, rare studies reported the chemotherapeutic regimens for treating bone metastasis. This may be an important research direction for bone metastases in the future. Recently, some novel treatments including molecular targeting, immunotherapy and stem cell therapy provide hope for the treatment of spinal tumors (10). Further studies are warranted to determine the novel treatment methods for those patients.

The current study still has certain limitations. First, this is a retrospective study with inherent bias. Second, detailed data regarding the radiotherapy and chemotherapy were unavailable. Third, the SEER database does not provide any information regarding local recurrence or distant metastasis during follow-up. Therefore, clinical randomized trials are urgently needed to verify our findings and improve the survival.

## Conclusion

Survival predictors for patients with bone metastasis of unknown origin included race, brain metastasis, liver metastasis, radiotherapy, and chemotherapy. Therefore, large range screening of the above independent risk factors can effectively improve the prognosis to a certain extent. Additionally, this study provides valuable reference information for clinicians and patients to make treatment decisions.

## Data availability statement

The raw data supporting the conclusions of this article will be made available by the authors, without undue reservation.

## Ethics statement

The studies involving humans were approved by Ethics Committee of the Second Affiliated Hospital, Zhejiang University School of Medicine. The studies were conducted in accordance with the local legislation and institutional requirements. The ethics committee/institutional review board waived the requirement of written informed consent for participation from the participants or the participants’ legal guardians/next of kin because clinical data in the present study were extracted from a public database with no patient identification info.

## Author contributions

ZW and NZ conceived and designed the study. YR, SQ, and GX collected the data. YR, SQ, GX, and ZC performed the statistical analysis. YR wrote the manuscript. ZW revised it. All authors contributed to the article and approved the submitted version.
